# A New Monitoring Effort for Asia: The Asia Pacific Mercury Monitoring Network (APMMN)

**DOI:** 10.3390/atmos10090481

**Published:** 2019-08-21

**Authors:** Guey-Rong Sheu, David A. Gay, David Schmeltz, Mark Olson, Shuenn-Chin Chang, Da-Wei Lin, Ly Sy Phu Nguyen

**Affiliations:** 1 Department of Atmospheric Sciences, National Central University, 300 Jhong-Da Rd, Taoyuan 32001, Taiwan; 2 National Atmospheric Deposition Program, Wisconsin State Laboratory of Hygiene, 465 Henry Mall, Madison, WI 53706, USA; 3 U.S. Environmental Protection Agency, Office of Atmospheric Programs, Clean Air Markets Division, 1200 Pennsylvania Avenue, NW, MC6204J, Washington, DC 20460, USA; 4 Environmental Protection Administration, Taipei 10042, Taiwan

**Keywords:** mercury, wet deposition, network, Asia, APMMN

## Abstract

The Asia Pacific Mercury Monitoring Network (APMMN) cooperatively measures mercury in precipitation in a network of sites operating in Asia and the Western Pacific region. The network addresses significant data gaps in a region where mercury emission estimates are the highest globally, and available measurement data are limited. The reduction of mercury emissions under the Minamata Convention on Mercury also justifies the need for continent-wide and consistent observations that can help determine the magnitude of the problem and assess the efficacy of reductions over time. The APMMN’s primary objectives are to monitor wet deposition and atmospheric concentrations of mercury and assist partners in developing their own monitoring capabilities. Network planning began in 2012 with wet deposition sampling starting in 2014. Currently, eight network sites measure mercury in precipitation following standardized procedures adapted from the National Atmospheric Deposition Program. The network also has a common regional analytical laboratory (Taiwan), and quality assurance and data flagging procedures, which ensure the network makes scientifically valid and consistent measurements. Results from our ongoing analytical and field quality assurance measurements show minimal contamination in the network and accurate analytical analyses. We are continuing to monitor a potential concentration and precipitation volume bias under certain conditions. The average mercury concentration in precipitation was 11.3 (+9.6) ng L^−1^ for 139 network samples in 2018. Concentrations for individual sites vary widely. Low averages compare to the low concentrations observed on the U.S. West Coast; while other sites have average concentrations similar to the high values reported from many urban areas in China. Future APMMN goals are to (1) foster new network partnerships, (2) continue to collect, quality assure, and distribute results on the APMMN website, (3) provide training and share best monitoring practices, and (4) establish a gaseous concentration network for estimating dry deposition.

## 1. Introduction

Mercury is a global pollutant that can be transported over long distances, across political boundaries and media. The cycling of mercury in the Earth’s environment is complex, and has many variables and processes occurring over time and space. Many studies have repeatedly shown the impacts of mercury on aquatic and terrestrial systems, including animals, plants, and people as mercury cycles in the environment. Given the global nature of this mercury problem and our limited understanding of these processes, work began on the Minamata Convention on Mercury in 2009 [1]. The Convention is an effort to fully understand and reduce anthropogenic mercury from entering the environment to protect human health and the environment. The Convention came into force on 16 August 2017 and currently, 128 countries are signatories to the Convention and 110 have ratified it.

Basic scientific measurements of mercury within different ecosystems and spheres is essential to this effort. Much of the environmental mercury today is emitted as an air pollutant. Many large and direct sources of anthropogenic mercury emissions have been reduced or eliminated throughout much of North America and Europe. The same processes of minimization and elimination are beginning around Asia. But it remains that in much of the world, the dominant pathway for new anthropogenic mercury input to ecosystems is through atmospheric emissions, transformation, transport, and deposition [2–11]. Therefore, it is particularly important to measure the movement of mercury from the atmosphere into the biosphere in order to track any improvement due to emissions reductions around the globe.

Atmospheric mercury can exist as many different compounds and so are typically treated as three different “fractions”, or primary forms in the atmosphere. These include: 1) gaseous elemental mercury, or Hg^0^ (GEM), 2) gaseous oxidized mercury, or mercury in the Hg^2+^ form (GOM), and 3) particulate-bound mercury (PBM). Most measurements are for PBM associated with particulate sizes less than 2.5 microns in diameter (PBM_2.5_). GOM is less volatile and more water soluble than GEM, and is likely to be removed by precipitation, or adsorbed onto exposed surfaces and atmospheric particles. Therefore, GOM and PBM are the primary atmospheric forms responsible for wet deposition of mercury in precipitation [9],but can also be important in dry deposition. More recent studies have suggested that the dry deposition of GEM is also quite important under many different situations [10–12].

Mercury moving from the atmosphere to the biosphere begins a process of biomagnification in food webs, leading to high mercury concentrations in higher level organisms and ultimately humans [13–16]. Human and wildlife exposure to mercury is primarily due to the consumption of contaminated fish [17], and others suggest that more recently emitted mercury is most readily converted to the organic and toxic form of mercury, methyl mercury, and is readily biomagnified [18,19].

In order to understand mercury cycling through the environment and bioaccumulation in food webs, mercury movement into the biosphere through atmospheric deposition is an important pathway to measure. Many regions of the world have established atmospheric mercury monitoring networks for repeated, long-term measurements; these include the continental-scale European Monitoring and Evaluation Programme [20], and the National Atmospheric Deposition Program of North America [21]. Several mercury deposition networks operate in Asia, such as the Mercury Wet Deposition Network of Taiwan [22,23]. Similar networks also operate or have operated in Korea, Japan, and China.

However, the lack of an Asian continental-scale network with accessible data and long-term measurements is an important and missing piece of the mercury puzzle, particularly for countries that have signed on to the Minamata Convention. Further, data suggest that anthropogenic mercury emissions are highest in Asia [15,24,25], and fish consumption is quite high in many of these countries [26].

Several studies have shown that atmospheric mercury emissions around the globe are either stable or increasing in recent years [27,28]. Generally, anthropogenic Hg emissions are decreasing in Europe and North America, but are increasing in East and Southeast Asia [27]. Decreasing trends in atmospheric GEM concentration have been observed at various sites including Cape Point, South Africa [29], and across Canada [30], among others. Decreasing atmospheric Hg concentration and Hg wet deposition has been observed at many sites in the U.S. [21,31], but recent increases in western U.S. wet deposition were noted.

However, long-term atmospheric Hg monitoring records in East Asia are more rare [25]. Wu et al. [32] estimated the anthropogenic Hg emissions in China during 1978–2014 and found that the emissions were decreasing after 2011. Kim et al. [33] combined monitoring records over 25 years in and around Seoul, South Korea, and reported total gaseous mercury concentrations decreasing significantly since the 1980s. Recently, Nguyen et al. [34] reported decreasing long-term trends of high elevation GEM concentrations at Mt. Lulin, Taiwan since measurements began in 2006. Marumoto et al. [35] measured decreasing GEM trends at Cape Hedo, Okinawa, Japan, but no significant annual trends in either GOM or PBM_2.5_. For deposition, Fu et al. [10] reported recent wet deposition rates in China from measurements and other studies. They report quite high precipitation concentrations and deposition rates of 52 ng L^−1^ and 56.5 ug m^−2^ year^−1^ in Nanjing, but with much lower values in other locations in China.

In consideration of these points, we began the discussion, design and development of a continent-wide network over the Asia region to measure the deposition rates of mercury compounds from the atmosphere. The result of the work is the Asia Pacific Mercury Monitoring Network (APMMN).

## 2. Development of the Asia Pacific Mercury Monitoring Network

The APMMN is a cooperative effort to systematically monitor mercury in air and precipitation throughout the Asia-Pacific region. The primary goal is to generate a database of scientifically valid measurements of mercury wet deposition and estimates of dry and total deposition over this region, while making the data available to each participating organization and to scientists and policy professionals worldwide. To fulfill this goal, we formalized several objectives:

- to make measurements of atmospheric mercury that would be used to determine the status and trends in concentrations of ambient mercury species for wet, dry, and total atmospheric deposition of mercury;
- to develop a robust dataset for regional and global modeling;
- to assist partner countries in developing monitoring and assessment capacity; and
- to distribute these data and information to all data users.

The development of a monitoring network can be quite challenging, particularly with costs of equipment, training of collaborators, and widely different operational systems between countries, among other issues. Our strategy was to first develop the capability to measure wet deposition among the different organizations and countries. After network establishment, our second step was to develop the capability to measure atmospheric concentrations of mercury from which dry deposition estimates could be made. Once this step was initiated, the network would move to build modeling and analysis capabilities which partner countries would need and use.

**Figure 1.**
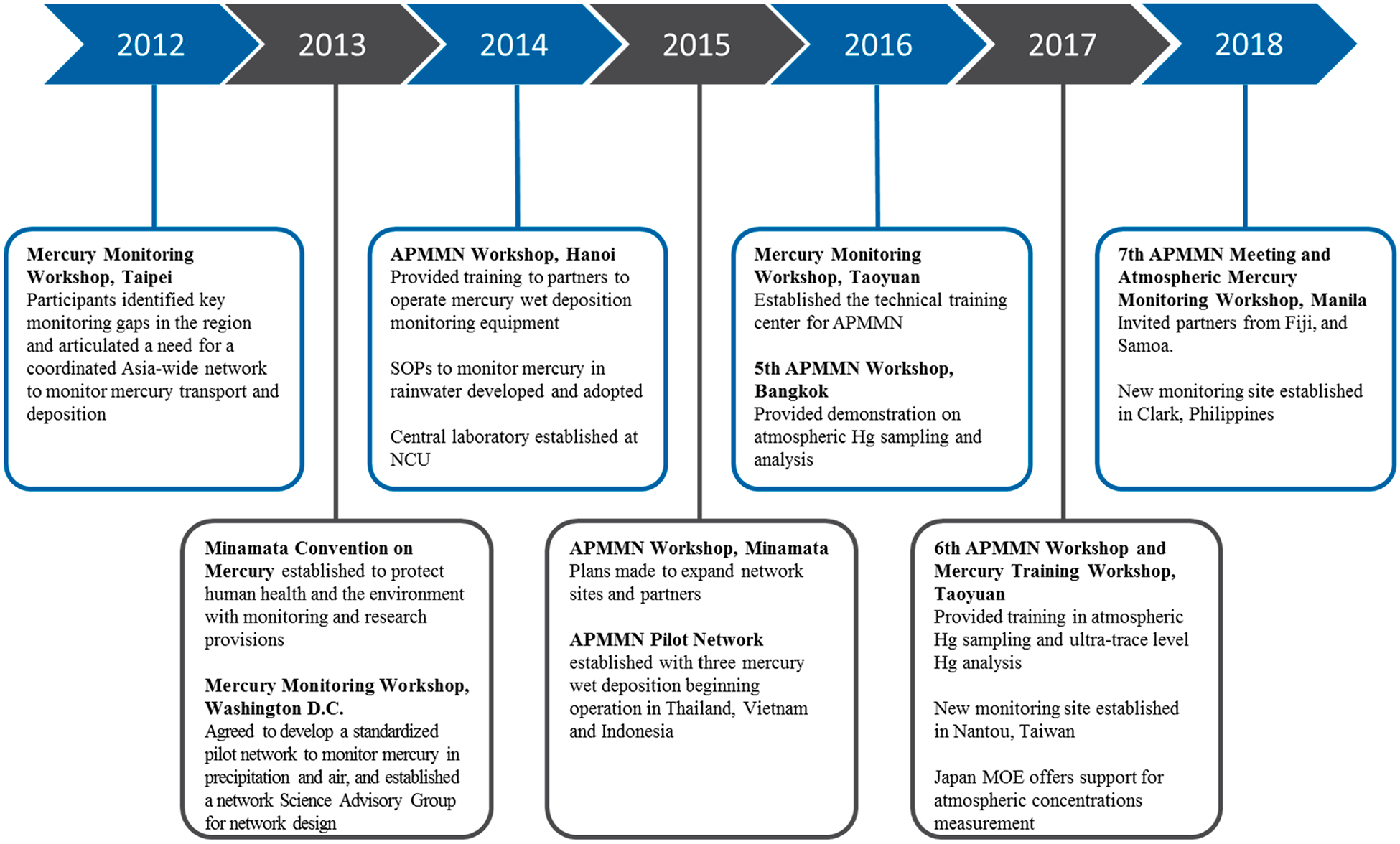
Timeline for the development of the Asia Pacific Mercury Monitoring Network, 2012 through 2018.

The initial development of the APMMN began in 2012 with a series of exploratory meetings with several different organizations in Asia (Figure 1). These initial meetings were supported by Taiwan’s Environmental Protection Administration (EPAT), the United States Environmental Protection Agency (US EPA), and by scientists from the National Central University (NCU) in Taiwan, and the National Atmospheric Deposition Program (NADP) in North America. Discussions centered around the key gaps in mercury information that existed in the Asia-Pacific region and the need for more information about the cycling of mercury, all of which to support the goals of the Minamata Convention on Mercury (which had not yet been ratified). We also introduced the idea of a coordinated continental network designed to meet the needs of everyone. After several meetings, an agreement among these several parties was made in 2013 to begin a coordinated and standardized trial network to monitor mercury in precipitation. A Scientific Advisory Group (SAG) was put together with membership representing EPAT, US EPA, NCU, NADP, representatives of the Ministry of Natural Resources and Environment (MONRE) in Thailand, the Vietnam Environmental Administration (VEA), the U.S. National Oceanic and Atmospheric Administration (NOAA), Environment and Climate Change Canada (ECCC), and others.

During 2014, the SAG invited several other organizations and countries in Asia to participate. The SAG developed standard operating procedures (SOP) for mercury wet deposition monitoring specific to regional conditions (little snow, heavy precipitation), while remaining consistent with other continental scale networks and their SOPs. A decision to centralize all network analysis into one laboratory was made following other networks, and that laboratory was established at NCU in Taoyuan, Taiwan. Operation of pilot sites was planned in Thailand, Vietnam, and Indonesia, with a quality assurance and testing site established in Taiwan at a national mercury wet deposition network site. Analytical SOPs were established for network use.

In 2015, several new partnerships were established, a network site liaison was hired to coordinate the activities among the network sites, and a network website was established for communication among members and to house the growing body of network documents, presentations, and meeting records. The pilot network began with the operation of three monitoring locations in Thailand, Vietnam, and Indonesia. The centralized analytical laboratory was expanded in 2016 by adding additional space, new instrumentation, permanent analytical staff, and additional quality assurance field measurements. NCU also established the Center for Environmental Monitoring and Technology. In part, the Center was established to support the work of APMMN as a central location for training, meetings, and network operation.

During 2017, new sampling equipment was delivered to several partner countries along with the addition of monitoring location in Taiwan. Also, plans for future atmospheric concentration measurements were advanced with a proposal by Japan’s Ministry of Environment (MOE) to support network atmospheric GEM concentration monitoring with a simplified gold trap method, and to support the monitoring with training. This additional capability will be taken up by those countries interested in further monitoring over the next several years. During 2018, a new monitoring location was established in the Philippines north of Manilla. APMMN also received a commitment to establish new sites in Sri Lanka, Fiji, Mongolia, and Nepal. Site establishment in these countries is in progress. Since the network was launched in 2013, annual meetings and training continue to occur at many locations throughout the region.

All of these developments have resulted in the current membership of APMMN being quite large and varied. Many different organizations are now involved with the network in some role; operation of one or more sites, financial support, scientific support, SAG membership, or potential support with future efforts such as the atmospheric concentration monitoring. The central organization is the National Central University in partnership with EPAT. NCU is providing both the centralized laboratory for consistent measurement and network coordination.

## 3. Methods Used by APMMN

Operation of the APMMN follows the general scientific consensus on the proper operation of wet deposition measurements for mercury. APMMN is also consistent with the SOPs of the NADP, EMEP, and those of Taiwan’s atmospheric wet mercury deposition network.

### 3.1. Collection Methods/Instruments

**Figure 2.**
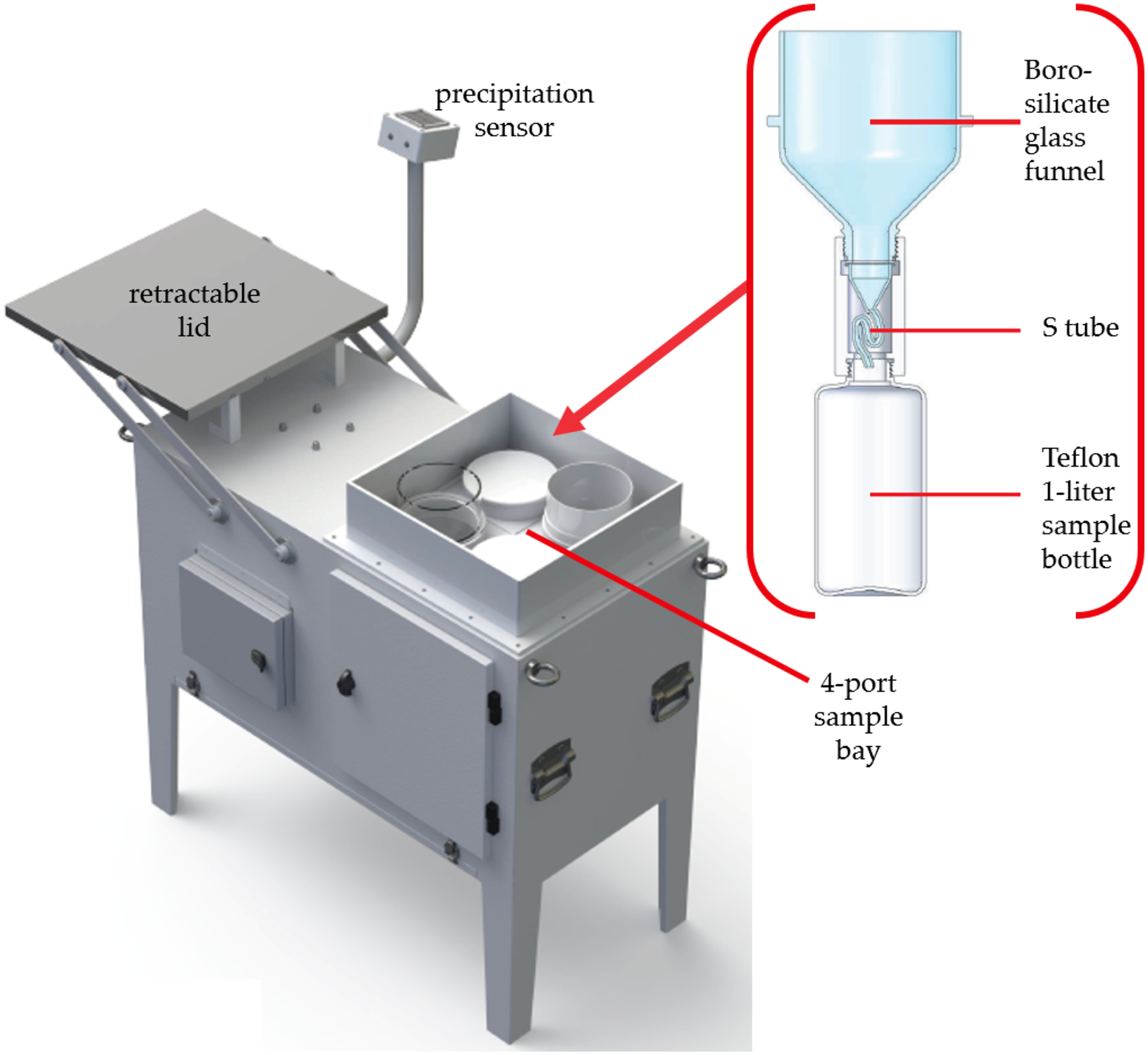
Asia Pacific Mercury Monitoring Network (APMMN) Wet Deposition Sampler, including the sample funnel and bottle.

Over the development of the APMMN, several wet deposition candidate collectors were used in the initial years, but one consistent sampler was always the goal for the network. Ultimately, the decision was made to use the model in use by Taiwan’s Mercury Wet Deposition Network, which is now being used by APMMN for all new sites and most of the original sites. The chosen sampler is the Mercury Deposition Sampler (Figure 2), manufactured by Fortelice International Co. Limited [36]. This sampler is a traditional wet deposition sampler and is very similar to the MIC-B sampler used by many Canadian and American researchers. The instrument has a painted, stainless steel body with an electronically controlled movable lid. The lid and action are controlled by a heated, optical precipitation sensor to signal the start of a precipitation event. The lid is controlled by a microcomputer programmable controller and motor, housed in an enclosure under the lid and the instruments sampling top.

Movement of the lid exposes or covers a 4-port sample bay where the precipitation collection is made. Four ports are available to make multiple samples during any precipitation event. Each port will hold a 140 mm glass funnel. The APMMN uses only one port, and the sample train consists of a borosilicate 140 mm diameter funnel connected to an “S-shaped” glass tube approximately 80 mm long used for evaporation control. The S-shaped evaporation tube is between a 1-L Teflon® bottle and borosilicate funnel for sample collection. The sample train is contained inside the body of the sampler to maintain integrity of the sample. The sample remains at ambient temperature during the sample period.

Due to the high cost of shipping sampling equipment, the APMMN has chosen to clean sample trains at each sampling site. The borosilicate funnel, S-tube glass connector, and sample bottles are cleaned onsite with 10% ultrapure HCl on a weekly basis. Training for glassware cleaning occurs at each training meeting. Additionally, bottle blanks from the sites are requested and analyzed by the laboratory regularly.

Clean sample bottles are precharged with 10 drops of trace metal grade 12-normal Hydrochloric Acid (HCl), following EPA’s Method 1631e [37] and methods of other networks. This precharge is used to ensure that the mercury collected will remain in solution and not be lost through vaporization (i.e., forced towards Hg^2+^ and away from Hg^0^). We have noticed that some of the different HCl sources are not appropriately clean of mercury, and have instituted a HCl testing program when the sampling results indicate contamination, and have flagged these sample results with possible contamination. Additionally, when the HCl was consistently of poor quality, no precharge was used for these samples. These biased samples are labeled as such in the database with appropriate data flags.

APMMN has an operational site SOP for all field and cleaning operations [38]. This SOP also includes a site selection procedure following the NADP guidelines and rules. Siting conditions are documented by each site and made available by APMMN. Field operators are trained on the SOP at each training meeting (annual) and upon installation of the sampler. The Field SOP follows the NADP SOP for sampling of wet deposition of mercury.

### 3.2. Field Operation

Each sample is an integrated 7-day sample, where all precipitation that occurs over that 7-day period is collected into one sample bottle (the typical length for deposition measurement). Samples are changed from 8 to 10 am local time each Tuesday, conforming to the basic global wet deposition sampling schedule. Each Tuesday a new, cleaned sample train is added to the sampler, with appropriate quality assurance used by the operators (gloves, technique, etc.). With each sample, the site operator completes a field sheet, including site name, operator initials, start and stop times, and potential issues with the sample (obvious contamination, site operation, etc.). This field sheet is returned to the laboratory with the sample. A new sample train is installed after removal of the current sample to ensure a continuous sampling for the entire year.

After collection of the sample, the sample train is returned to the site laboratory. The collected sample is poured from the sample bottle to a 150 mL polyethylene terephthalate glycol (PETG) sample transfer bottle to be shipped to the Central Laboratory. The sample is shipped to the laboratory at environmental temperatures. It should be noted here that bromine monochloride (BrCl) is not added to the sample bottle before pouring to the 150 mL sample transfer bottle. This deviation from EPA Method 1631e [37] was made due to the high oxidation potential of BrCl and the concern for operator safety at our sites, and a transfer bottle was chosen to minimize the very high cost of shipping in Asia. This change could result in a negative bias for the results. However, Parker and Bloom [39] found that with a 10.0 ng mercury L^−1^ concentration in natural waters with acid precharge, as we have, there would be between 3 and 8% of mercury loss to the walls of the original sample bottle. We deemed this an acceptable bias to our sampling at the beginning of our network. Addition of BrCl will occur in the network in later months as the APMMN matures.

### 3.3. Analytical Methodology

All network samples from affiliated sites are sent to NCU for total mercury analysis in precipitation. The lab is outfitted with two level 1000 clean rooms, three Tekran 2600 Cold Vapor Atomic Fluorescent Spectroscopy (CVAFS) Analyzers, and two separate clean benches. Analysis of sample concentrations follows the methods developed over time and standardized as US EPA Method 1631e [37]. This method utilizes elemental mercury capture on gold traps, followed by Cold Vapor Atomic Florescence Spectroscopy for detection of total mercury as elemental mercury. The network’s laboratory uses the Tekran Model 2600 Automated Sample Analysis System for consistent measurement of the networks wet deposition samples [40]. The method detection limit is 0.2 ng L^−1^ and a minimum level of quantification of 0.5 ng L^−1^.

The network runs several quality assurance checks over the course of routine sampling, including system blanks, bottle blanks, duplicate analyses, Certified Reference Material (CRM, source ORMS-5 from the National Research Council Canada and used for all periods), and matrix spikes. Results from these analyses are presented in the next section.

### 3.4. Data Planning, Release of Observations

After analysis, field and laboratory data is stored in a secure NCU database. Observation from the field records are added to the database, along with the observed analytical concentrations, etc. Any laboratory quality assurance issues are also recorded. Based on the field and laboratory results, each sample is given a quality code (A, B, C = invalid), following the same rules as NADP and Taiwan’s network. Specific conditions resulting in valid and invalid samples are shown in the SOP. Results are shared with the sites for a completeness and accuracy review. Currently, sites have the option of releasing their data publicly. Some sites have chosen to do this, while others have chosen not to release their site data. However, it is the goal of the network for full release of all data soon.

## 4. Results

As of the time of this writing, some results from the network can be shared. The first information to consider is the network information available at the APMMN website [41]. The website has been operational since 2015, and contains an abundance of information. Details of APMMN’s development are listed here, along with the annual meeting details, presentations, and photos. A map of the current operating sites, with siting information and photos are available. The SOPs are also available here and are downloadable for use by others. Additional information is provided about our training program, along with individual training videos. Finally, contact information for the network is also provided.

### 4.1. Current Sites

**Figure 3.**
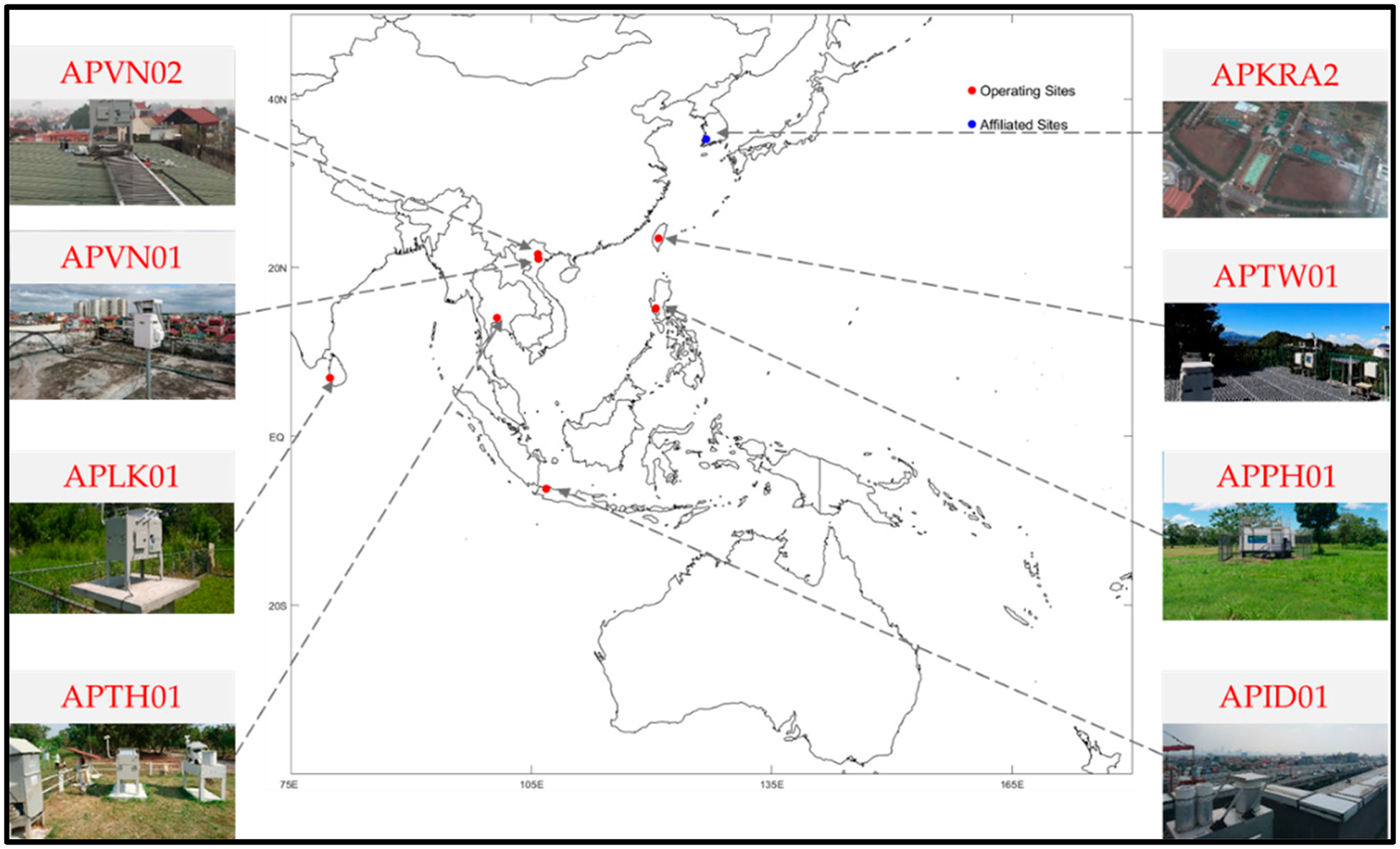
Currently monitoring sites of the Asia Pacific Mercury Monitoring Network, as of 2018.

The currently operating APMMN sites are showing in Figure 3. Seven stand-alone sites are currently sending in samples, and include sites in Thailand (suburban Bangkok), Indonesia (Jakarta), Philippines (suburban Manilla), Sri Lanka (Un. of Peradeniya, near Kandy), Taiwan (Mt. Lulin) and two in Vietnam (Hanoi, Thai Nguyen). One affiliated site is in Gwangju, South Korea (Gwangju Institute of Science & Technology). In addition to these sites, several other sites will have equipment and hopefully start monitoring during 2019. These sites include a site in Katmandu, Nepal; a site on Viti Levu Island of Fiji; a second site in Indonesia; and a site near Ulaanbaatar, Mongolia. Equipment for these new sites have been secured and installation is awaiting approval. Specific information about the operating sites is detailed in Table 1.

Additionally, scientist from both Japan and Korea have helped advance the APMMN effort. In Korea, one affiliated scientist is working with APMMN and providing sampling at our APKRA2 site (Gwangju Institute of Science and Technology). We are working with other partners in Korea to share best monitoring practices and align operations with the Korean national network, where possible. Several organizations in Japan have been instrumental in the network development, providing both scientific support, equipment, and expanding atmospheric measurements as part of a future network (see the future plans section).

For network operation, it is always important to run a centralized quality assurance site within the network. For the APMMN, we are operating a multi-sampler site at the NCU laboratory. In addition to the operating network site, the APMMN is also operating a second MIC-B style collector (a duplicate of the network sampling site), and an NCON Wet Deposition sampler (one in network, also the NADP approved sampler). Results from these duplicate samplers provide both quality assurance information about sampler variability within the network, and comparability information to other networks. Results from these quality assurance samples are provided in the following sections.

### 4.2. Current Quality Assurance Results

Quality assurance results from the network and laboratory are presented here.

#### 4.2.1. Bottle Blanks, Duplicates, Reference Comparisons

The network quality assurance results are shown in Table 2. Thus far, results from the APMMN network are very good, and consistent with those of other networks. The results are divided into two date ranges, given that a new instrument was integrated into network service in November 2017.

System blanks are analyzed to demonstrate that the analytical system is free from contamination at levels that could affect data quality. Results show that the system blanks in all years are quite low, and significantly lower in 2018 which shows improvement with time. These concentrations are on average approximately two orders of magnitude below concentrations seen thus far in the network (0.07 (+0.02) ng L^−1^). For 2018, the maximum measured is quite reasonable, and much lower than in earlier years. These results suggest that there is little total contamination in the analytical system.

Duplicate sample analyses were run periodically, and the results show very low differences between duplicates over 190 analyses. Typical measures for 2018 are less than 3% difference between measurements. The largest difference was reported at 11% (see Table 2).

For matrix spikes, where the laboratory adds a known amount of mercury to a precipitation sample, the sample recovery ranged from 83.7% and 119.6% over all years, and between 83.7 and 104.6% in 2018, which we deem to be an acceptable range for such low concentrations, and in line with typical measurement laboratories. On average, we currently recover 98.1% of the mercury.

The APMMN also runs Quality Control Samples. These are prepared from a certified mercury solution with a source different from the one used to produce the standards run routinely during sample analysis. These are analyzed as an independent check of system performance. For the 122 samples run thus far, our analysis agrees with the known concentration within 1% over all years, and averages 99.8% of the value in 2018. The largest difference seen this far was −8.3% below the expected result.

Additionally, for our analysis versus a Certified Reference Material standard (Source ORMS-5), the laboratory typically recovers 94% of the standard, showing a small negative bias here, but consistent with general laboratory practice.

Overall, APMMN analytical laboratory results to date have been very reasonable. We are demonstrating that our glassware is clean, our samples are not being contaminated by storage or shipping, and APMMN is accurately determining the concentrations of the wet deposition concentrations for mercury in the network.

#### 4.2.2. Sampler Type Comparisons

As noted before, the majority of samplers in the network are MIC-B style collectors. However, one of the samplers is an NCON Wet Deposition Collector (NCON Systems, Crawford, Georgia, USA) as used in the NADP’s MDN network. Therefore, we have made some basic comparisons of the different samplers on concentration and sample volume measurements collected at the NCU campus in side-by-side collection.

**Figure 4.**
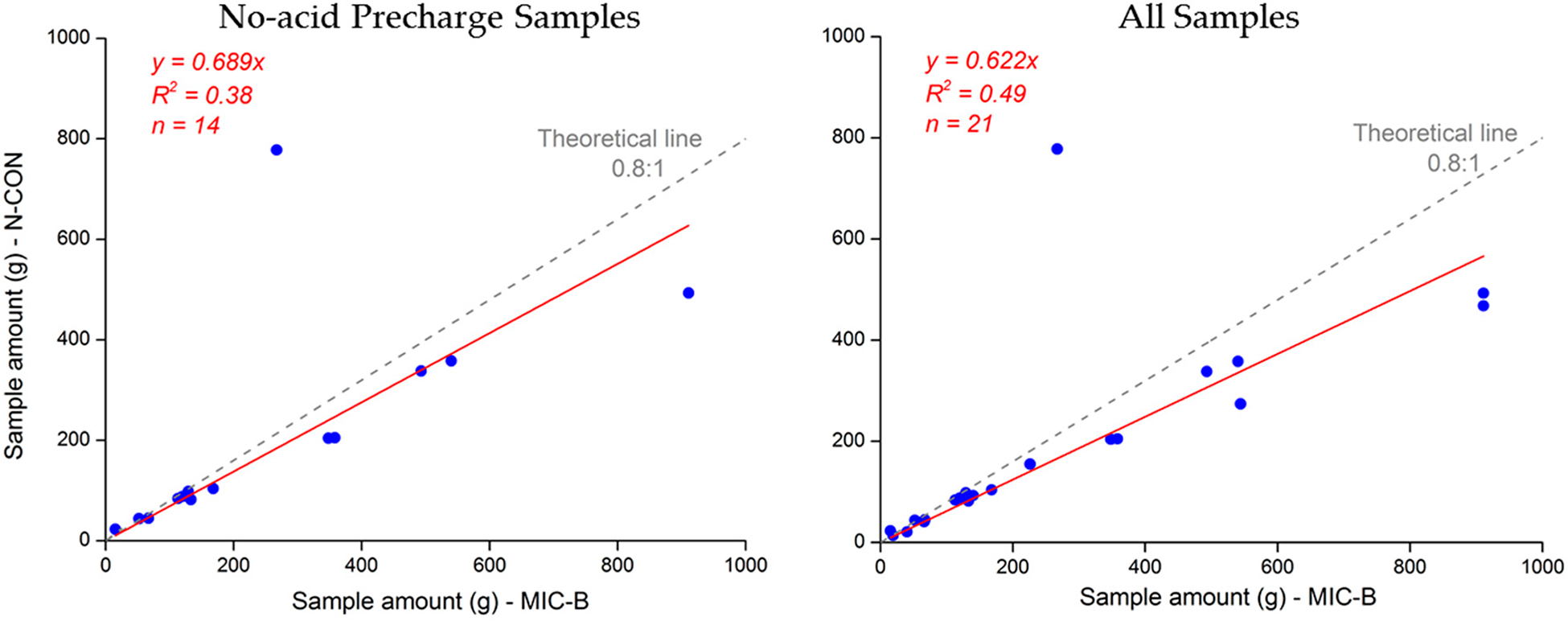
Volume collected by collocated samplers using two separate sampler types (NCON and the MIC-B Style) at National Central University (NCU) Campus, Taiwan. Acid precharge sample volumes for both samplers do not include acid volume added.

In Figure 4, some of these comparison results are shown. For the volume comparison, note that the NCON funnel is 125 mm in diameter versus a 140 mm diameter funnel with the MIC-B style funnel. Therefore, with 80% of the MIC-B style funnel area, a low bias of precipitation collection is expected for the NCON. The 0.8 to 1.0 line (area relation) is shown. The graphic on the right is for samples from both samplers with and without acid precharge together (20 mL 0.12-normal HCl), and the graph on the left is for samples from both samplers without acid precharge only. We also removed 4 samples where weekly precipitation exceeded 60 mm due to overflow of the sample volume collection and the introduction of another unknown. We measure a significant bias towards lower volume captured in the NCON collector of about 11% without any acid addition. With acid addition, the bias is higher at 18%. This result is a surprising one; we expected to see some differences, as noted by other researchers [42]. But volume biases on this level were unexpected between the two different collectors. Also, the addition of acid precharge to the sample seems to increase the precipitation differences with even less precipitation collected by the NCON, although this increase is small (5%).

We are continuing to study these differences, but at this point we feel there can be several causes of this difference. Overall, subtropical heating of samples should increase evaporation of both collectors over the evaporation of collectors in the midlatitude networks. Additionally, these collector volume differences could have three different explanations: (1) missed NCON precipitation most likely at the beginning of the rain event, (2) precipitation hitting nearby surfaces and bouncing into the MIC-B style funnel (so called precipitation bounce), or (3) additional evaporation by the NCON sampler over the MIC-B style sampler.

For the first explanation, both collectors use laser based optical sensors and open when *n* droplets of precipitation fall through the beam. If the NCON is opening late and missing early precipitation, then this effect would get smaller with higher levels of precipitation (1 inch of precipitation is approximately 390 g of MIC-B style sample). This is not the case with the samples we have thus far; the volume difference remains pronounced at the high and the highest precipitation amounts. Also, we fail to see how the addition of acid would make this issue worse. Therefore, we are skeptical of this explanation.

For the second explanation of MIC-B style bounce on the relatively large surface near the opening, the MIC-B style clearly has a large surface area for potential precipitation bounce. The NCON collector was designed to minimize the possible bounce surface area. Also, every sizable precipitation event (save the outlier at 800 g NCON) is biased towards more precipitation in the MIC-B style collector. However, at higher precipitation we would expect this bounce effect to increase the amount collected and increase the percentage differences. We also see no reason why adding acid would increase this effect. We therefore must keep this explanation open, at least at this time.

For the third explanation, the traditional connection for the NCON funnel-to-bottle (and used by the MDN in NADP) is a long small diameter tube with a blown bulb that sits in the mouth of the sample bottle to control evaporation, rather than a screw and trap sample train as described here. With this arrangement, there is a non-airtight connection for the NCON sample train, and reason to suspect added evaporation under the very warm conditions in Taiwan. Further, with added acid, a possibility exists that the acid volume may evaporate more quickly than water, all supporting the observations seen in Figure 4.

If you include the overflow samples in this analysis (not shown), the percentage of negative bias associated with the NCON goes to zero for samples with no acid addition. With acid addition, the negative bias increases by −4%. We feel that the added complication of overflow sample likely masks the volume differences and only complicates determining the cause. So we consider these observations less important to this issue. However, the overflow results tend to support the evaporation driven differences (exp. 3).

At this point, explanation 3 must be considered the leading explanation for the bias in NCON sample volume. Further tests are planned and will be reported in the future.

**Figure 5.**
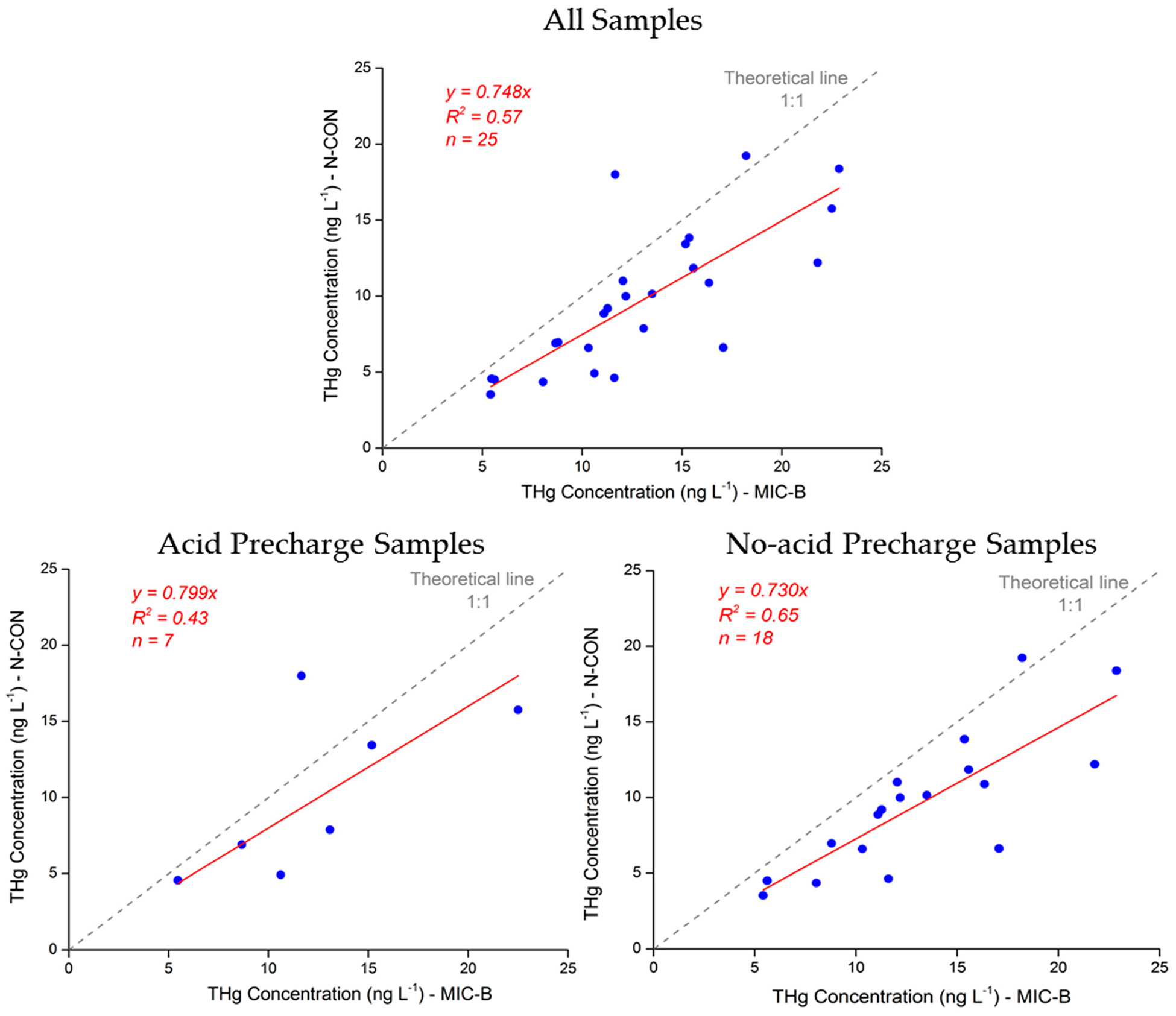
Concentrations measured by collocated samplers using two separate sampler types (NCON and the MIC-B style) at NCU Campus, Taiwan.

Another important test is the test for mercury concentrations differences between sampler types, which are shown in Figure 5. The results do show a significant negative bias present for the NCON sample and sample train. The MIC-B style sampler concentrations are about 25% higher with both acid precharge and non-precharge samples (Figure 5, upper figure). The precharged samples (Figure 5, lower left) and non-precharged samples (Figure 5, lower right) vary a bit, with the non-precharged samples having a slightly higher bias (7%).

What are the explanations for these observations? We think there are several possible explanations for these concentration differences in precipitation. If the concentration differences were large with only low volume/high concentration samples, this would suggest that the NCON sampler was not opening early during a rain event and missing the initial very high concentration droplets. But this does not seem to be the case; concentration differences are large even in heavy rain weeks where this type of effect would be diluted away.

A more likely explanation is evaporation of mercury from the deposited precipitation with the NCON. This explanation is strengthened somewhat by the NCON sampler and sample train evaporative issues noted previously. There may be an evaporation issue, and it follows that mercury could be lost too. The acid addition subset of observations suggest that the loss of mercury concentration is lessened with the addition of the HCl, which is used to stop loss of mercury. Based on this small number of acid-added samples (7), we would have to conclude the acid addition does retard the loss of mercury but does not seem to retard all mercury losses. More observations of these tests will be needed to confirm these observations.

Comparisons of the different network samplers will continue, and additional observations will be published in the future.

In comparison of the two co-located MIC-B style network samplers operating at the NCU campus, we do see some marginal differences between the MIC-B style collectors. But these small differences are consistent with other samplers and networks [42]. Overall, the collocated MIC-B style collectors agree in terms of collected volumes and mercury concentrations quite well. APMMN will continue these within sampler type comparisons into the future.

In general, there are substantial differences in the NCON and MIC-B style samplers seen at our quality assurance site, and we will continue to monitor and report these observations. Therefore, with these observations, the APMMN needs to be careful when comparing our results to other network observations, at particularly with the one NCON collector in our network.

### 4.3. Field Results

At the start of operation, the APMMN members were not yet willing to freely release the concentration data without further review and approvals within their individual organizations. Each site and organization is free to release the data as they see fit. The APMMN has agreed to not release data until the members vote for full release. This full release has yet to occurred, so we are only able to release general observations from the field measurements.

During the 2018 calendar year, the APMMN sites made 139 wet deposition measurements over SE Asia. For all of these measurements, the average wet deposition of mercury was 11.3 (+ 9.6) ng L^−1^. Given this deviation about the mean, concentrations varied widely during the year and over space.

APMMN is recording highly variable concentrations within most sites. Average concentrations for the individual APMMN sites vary from about 6 ng L^−1^ per year to about 35 ng L^−1^, which is quite a large range over southern Asia. As in many networks, high average concentrations are not limited to the urban sites, but vary across the urban/suburban/rural gradient. We also note some sites with very high standard deviations about the mean; on the order of +15 ng L^−1^ at some locations, with other sites showing very low standard deviations (+5 ng L^−1^).

Clearly, the measurements made thus far show that some sites have very low concentrations, comparable to the measurements made in the Northeast United States (both urban and industrial), along the West Coast of the United States [21,31], and comparable to the lower concentration measurements made at Pengjiayu, Taiwan [22]. Annual precipitation weighted concentrations across the U.S. range from 2.0 to 25.0 ng L^−1^, depending upon the year. Other sites have relatively similar concentrations to the average condition seen in North America [21,31]; this average is approximately 9.75 ng L^−1^ in 2009. However, it is also the case that some of the initial observations show averages well above the highest North America concentrations measured along the Gulf of Mexico, and well above the range reported in Taiwan and in Europe. In one instance the concentrations compare favorably to observations reported in several urban centers in China, such as Chongquin (30 ng L^−1^) and Nanjing (52 ng L^−1^) [43,44].

With more evaluation of quality assurance, more sample periods, and clear multi-year measurement over the monsoon and non-monsoon periods, we fully expect that these initial indications could change. With the appropriate member approval, a release of the observations of mercury deposition will occur in the near future.

## 5. Future of the APMMN

Clearly, the APMMN is just beginning its measurement period. Although APMMN is new, we are planning for a long period of operation and measurement. In addition to the successes we have had thus far and have described here, we have also begun to make plans for the next phases of the of the network. We note a few of our five-year goals here.

- Excellent coverage over all of Asia and the Pacific region. To fulfill this goal, we hope to have 20 sites operating, with at least one site in all countries if possible.
- All APMMN data measured would be publicly available. This is a member decision, but we will work towards this goal.
- Since wet deposition is only part of total atmospheric deposition moving into ecosystems, we plan to work with our partners to systematically measure atmospheric concentrations across the Asia-Pacific region to complement the wet deposition measurements.
- Following from this goal, we will also work to develop a modeling methodology to estimate dry deposition fluxes using APMMN gaseous measurements.
- We would like to formally work more closely with the other mercury networks, in support of the Minamata Convention. These networks would include NADP, EMEP, the individual networks in Asia, etc. to advance globally comparable monitoring data.
- We plan to continue to have the highest of quality assurance in all of our network operation and measurement. Over the next five years, we will improve our methods and data review to continue the highest quality assurance possible and report these values appropriately.
- We plan to continue our network training in all network activities, and to develop additional training programs for the mercury related activities that our membership requests. We envision that the APMMN will contract with experts in these areas and arrange for training sessions at the NCU Training Center.

As the APMMN matures, some of these goals could evolve, but they are a good summary of the direction we feel appropriate for the network.

## 6. Summary

Given the lack of a continental-scale mercury network in Asia and the Pacific region and the immediate need for more measurements in support of the Minamata Convention on Mercury, several organizations and scientists gathered in 2012 to discuss these issues. From this meeting and several more, the Asia Pacific Mercury Monitoring Network was developed to provide consistent and continental-scale network measurements of the wet deposition of mercury.

Through the intervening years, the APMMN has grown to a current eight operating sites measuring weekly wet deposition of mercury across the Asia-Pacific region. These sites include locations in Thailand, Indonesia, Philippines, Sri Lanka, Taiwan, two in Vietnam, and South Korea. In addition, several other sites should start during 2019, and include locations in Katmandu, Nepal, Viti Levu Island in Fiji, a second site in Indonesia; and a site near Ulaanbaatar, Mongolia. The APMMN began sampling in 2015, and includes a centralized analytical laboratory, SOPs, a standard sampler and sample train (at almost all sites), a website for communications and data release, and regular annual meetings complimented with operator training.

Operational quality assurance is an important part of the network, and we reported initial results here. We have a full complement of ongoing analytical and field quality assurance measurements that we make. Results for our bottle blanks assure minimal contamination in the network, while the duplicates and standard comparisons show that our analysis is accurate and compares favorably to other networks. For field quality assurance, APMMN has multiple MIC-B style collectors operating at our Taiwan site, so we are able to measure the sampler variability due to equipment differences. Sampler differences are present, but are also typical for wet deposition networks. A second sampler (NCON) shows additional variability in measured volume and concentration. This sampler is only at one site, so this variability is limited. The collectors do show evaporative losses, which we are undertaking further measurements to determine completely.

The concentrations measured in the APMMN are still restricted by the member countries, so we have few concentrations to release at this point. However, overall, 2018 concentrations average 11.3 (± 9.6) ng L^−1^ for 139 samples. Some sites have very low concentrations, comparable to the very low West Coast samples of the United States, while other sites have average concentrations similar to high values being reported from many urban areas in China.

Although the APMMN is essentially just beginning its operation, the goal is to operate for many years into the future to clearly define deposition patterns over Asia and the Pacific region, and determine any trends in the mercury wet deposition. Several additional goals for the network over the next five years, include full and free data release for the wet deposition weekly observations, the development of an approach for measuring atmospheric concentrations of GEM, GOM and PBM_2.5_ and modeling of dry deposition based upon these concentrations, and full coverage of the Asia Pacific region with both wet deposition and gaseous mercury measurements.

Finally, the APMMN is a monitoring cooperative that includes many separate individuals and organizations over Asia and the Pacific region. We will continue to build capacity among these organizations to improve their ability to make mercury measurements in a scientifically valid manner, and for topics beyond deposition that the members consider important in their scientific and policy plans.

## Figures and Tables

**Table 1.** Monitoring site information for Asia Pacific Mercury Monitoring Network sites, as of 2018.

Country	Site ID	Site Name	City	Latitude	Longitude	Elev. (m)	Status	Type	Sampler
Indonesia	APID01	MOEF	Jakarta	6.233 S	106.877 E	24	Active	Wet	AEROChem
Korea	APKRA2	GIST	Gwangju	35.228 N	126.841 E	33	Active	Wet	NCON
Philippines	APPH01	Clark	Pampanga	15.177 N	120.536 E	184	Active	Wet	MIC-B style
Sri Lanka	APLK01	U of Peradeniya	Central Province, near Kandy	7.252 N	80.595 E	481	Active	Wet	MIC-B style
Taiwan	APTW01	Lulin	Nantou	23.469 N	120.873 E	2862	Active	Wet/ Gaseous	MIC-B style
Thailand	APTH01	ERTC	Pathum Thani	14.046 N	100.714 E	6	Active	Wet	MIC-B style
Vietnam	APVN01	CEM	Hanoi	21.049 N	105.883 E	16	Active	Wet	NCON
Vietnam	APVN02	Thai Nguyen	Thai Nguyen	21.584 N	105.840 E	31	Active	Wet	MIC-B style

**Table 2.** Asia Pacific Mercury Monitoring Network quality assurance results from two periods, 2016–2017 and 2018. A new analysis instrument was added at the end of 2017.

Quality Assurance Metric	Date Range	N	Mean (ng L^−1^)	Minimum (ng L^−1^)	Maximum (ng L^−1^)
System Blanks	2016–2017	127	0.18	0.05	0.53
	2018	51	0.07	0.03	0.15
Duplicate Analyses Differences			(%)	(%)	(%)
	2016–2017	128	1.0	0.0	4.9
	2018	62	2.7	0.0	11.2
Matrices Spikes	2016–2017	130	101.5	96.5	119.6
	2018	32	98.1	83.7	104.6
Quality Control Samples	2016–2017	83	100.5	95.6	106.3
	2018	39	99.8	91.7	105.5
Certified Reference Material	2018	8	94.0	88.5	98.4
